# Children's Hospital for Johannesburg

**Published:** 1920-08-28

**Authors:** 


### Children's Hospital for Johannesburg.

When Mr. Julius Jeppe presided over a meeting of the General Committee of the Children's Hospital for Johannesburg, the report showed that the appeals for help in the movement to build and equip a hospital in Johannesburg for the use of the children of the Transvaal, as a thankoffering for the conclusion of the war, had met with an encouraging response. The Johannesburg Town Council had granted a piece of ground, approximately eight acres in extent, close to the Queen Victoria Maternity Hospital. A generous gift of £2,000 had been received from the Central Mining and Investment Corporation, Ltd.; the Governor-General's War Relief Fund had given the sum of £5,000 for a ward to be called the " South African Gifts and Comforts Ward "; and the South African Red Cross Society had contributed £5,000 to establish a ward to be named the " Red Cross South Africa Ward." It was stated that the school children of the Transvaal are taking up the matter, and through the co-operation of the various Teachers' Associations, every child of school age will have an opportunity of contributing. The hospital will be available for children from all over the Transvaal Province.

The South African Trained Nurses' Association held a delightful ball on June 30 in aid of the Fund. The Overseas Club, the Martha Washington Club, and the Women's Enfranchisement League have promised help, and each one hopes to establish a bed at a cost of £500 which shall perpetuate the name of the owner.

